# The Florida Sleeve Procedure Is Durable and Improves Aortic Valve Function

**DOI:** 10.1055/s-0039-1687854

**Published:** 2019-09-17

**Authors:** Seyed Hossein Aalaei-Andabili, Tomas D. Martin, Philip J. Hess, Ashkan Karimi, Anthony A. Bavry, George J. Arnaoutakis, Thomas M. Beaver

**Affiliations:** 1 Division of Thoracic and Cardiovascular Surgery, Department of Surgery, University of Florida, Gainesville, Florida; 2 Division of Cardiology, Department of Medicine, University of Florida, Gainesville, Florida; 3 Division of Thoracic and Cardiovascular Surgery, Department of Surgery, Indiana University, Indianapolis, Indiana; 4 North Florida/South Georgia Veterans Health System, Gainesville, Florida

**Keywords:** Florida Sleeve, safety, aortic insufficiency, freedom from reoperation

## Abstract

**Background** The Florida (FL) Sleeve procedure was introduced as a simplified approach for valve-sparing correction of functional Type I aortic insufficiency (AI) associated with aortic root aneurysms. In this study, short- and long-term outcomes after the FL Sleeve procedure were investigated.

**Methods** From May 2002 to January 2016, 177 patients underwent the FL Sleeve procedure. Left ventricular end-diastolic diameter (LVEDD), left ventricular end-systolic diameter, left ventricular ejection fraction, and degree of AI (none = 0, minimal = 1, mild = 2, moderate = 3, severe = 4) were evaluated by echocardiography.

**Results** Mean ± standard deviation of age was 49.41 ± 15.37 years. Survival rate was 98% at 1 year, 97% at 5 years, and 93% at 8 years. Freedom from reoperation was 99% at 1 year and 98% at 2 to 8 years. Three patients (1.69%) died during hospitalization. Three patients (1.69%) developed periprocedural stroke. Postoperative follow-up echocardiography was available in 140 patients at 30 days, and 31 patients at 5 years. AI grade significantly improved from baseline at 30 days (2.18 ± 1.26 vs. 1.1 ± 0.93, *p* < 0.001) and at 5 years (2.0 ± 1.23 vs. 1.45 ± 0.88, *p* = 0.04). Preoperative mean LVEDD significantly decreased from 52.20 ± 6.73 to 46.87 ± 8.40 ( *p* < 0.001) at 30 days, and from 53.22 ± 7.07 to 46.61 ± 10.51 ( *p* = 0.01) at 5 years.

**Conclusions** The FL Sleeve procedure is a safe, effective, and durable treatment of aortic root aneurysm and Type I AI. Long-term survival and freedom from reoperation rates are encouraging.

## Introduction

Aortic valve replacement (AVR) and ascending aorta replacement were the standard approaches for the management of aortic root aneurysm combined with aortic insufficiency (AI). 1 AVR with a bioprosthetic valve increases the risk of reoperation, and AVR with a mechanical valve is associated with anticoagulant-related hemorrhage. 2 Later, two aortic valve-sparing (AVS) approaches (Yacoub and David 3 4 ) were developed to preserve native aortic valves to eliminate lifelong anticoagulant therapy. Although excellent results of these two AVS approaches were documented, 5 6 most centers' adoption of the Yacoub and David AVS procedures has remained at 15% because of their complexity, and many centers still consider AVR as first-line treatment. 1 7 The Florida (FL) Sleeve technique was introduced at Shands Hospital at UF Health (University of Florida, Gainesville, FL) to simplify the procedural complexity and preserve the native aortic valve in patients with AI secondary to aortic root dilation. 8 The FL Sleeve procedure does not require coronary artery reimplantation, which decreases the risk of surgical bleeding. 8 9 Moreover, the most recent study comparing biomechanical characteristics of the FL Sleeve and previous AVS techniques found that the FL Sleeve technique is even superior to previous AVS techniques in biomechanical standpoint as it leads to lower aortic valve stress and prevents possible aortic root distortion or harmful aortic wall stresses. 10 Hess et al 8 9 and Gamba et al 11 have reported encouraging early and midterm outcomes of the FL Sleeve procedure. In contrast to large studies incorporating David and Yacoub techniques, 5 7 12 all prior reports about the FL Sleeve procedure have included limited numbers of patients and reported early or midterm outcomes. 8 9 The long-term survival rate, freedom from reoperation, aortic valve function, and dimensional stability of the left ventricle following the FL Sleeve procedure remain unknown. Thus, to address these gaps of knowledge, we investigated outcomes of all patients who underwent the FL Sleeve procedure at our center.

## Materials and Methods

### Study Population

In this retrospective and single-center study, all patients with AI secondary to aortic root aneurysms who underwent the FL Sleeve procedure at Shands Hospital at UF Health (The University of Florida College of Medicine, Gainesville, FL) were included. The study was conducted from May 1, 2002 to January 1, 2016 after the Institutional Review Board approval and waiver of informed consent. However, patients who had the FL Sleeve procedure after March 2006 were consented, allowing us to follow up patients' status and clinical information through their primary care provider (PCP) and cardiologists. Our inclusion criteria were patients with normal or slightly abnormal leaflets and Type I AI secondary to aortic root aneurysm. Patients were excluded from the study for any of the following reasons: severely damaged, prolapsed, or nonfunctional aortic valve leaflets that needed AVR and patients with Type 2 AI.

### Procedural Technique

**Fig. 1:**
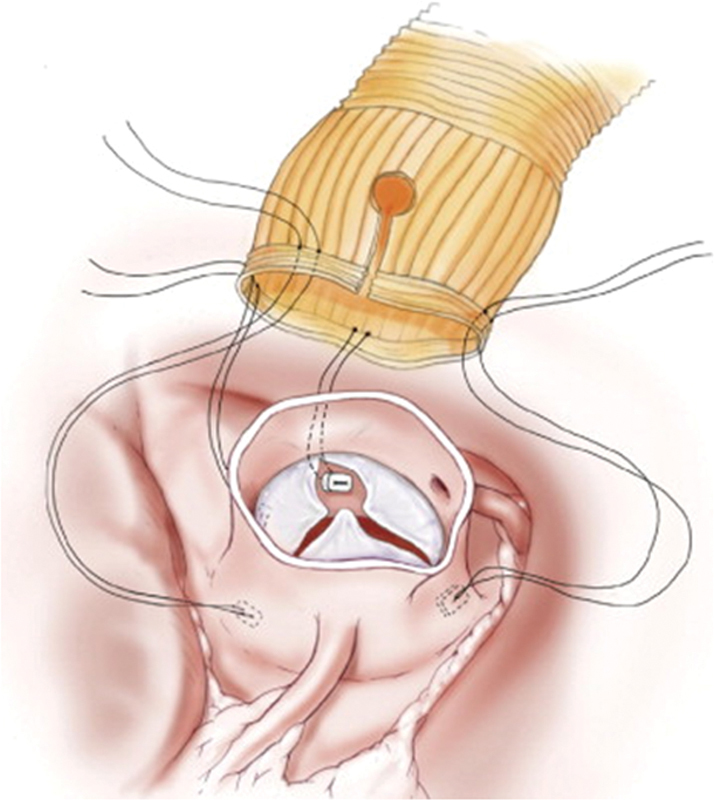
The four subannular anchoring sutures are placed in the same horizontal plane, 2 to 3 mm below the lowest point of the center of the leaflets; three are in line with the commissures, and the fourth is placed under the noncoronary cusp. The left coronary artery keyhole is cut after the sleeve is temporarily seated. The slits in the graft below the coronary keyholes are repaired after the sleeve is seated.

**Fig. 2:**
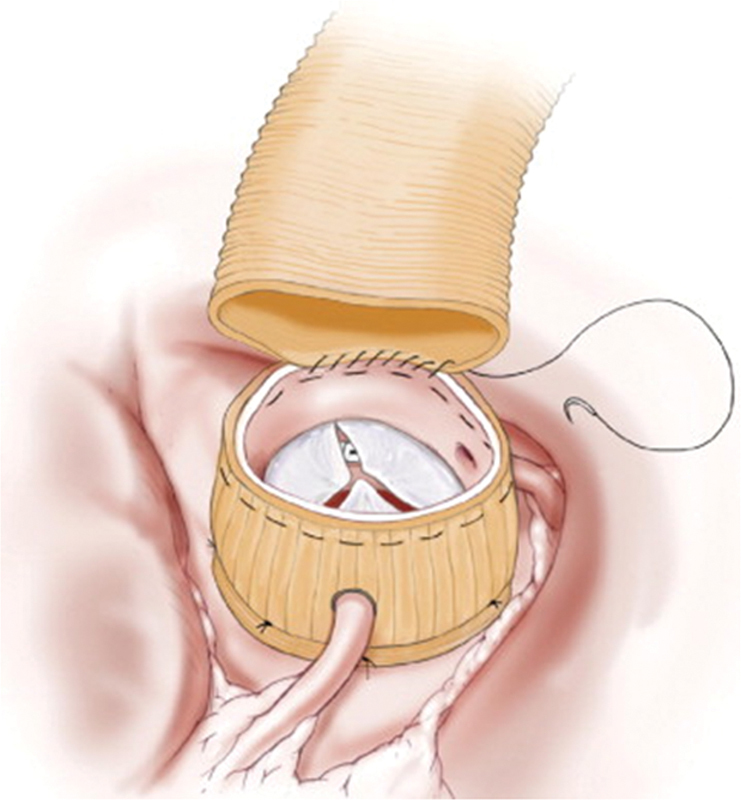
The running horizontal mattress suture both suspends the aorta and orients the posts of the commissures. Redundant aortic wall at the sinotubular junction should be imbricated with small pleats using multiple, closely spaced bites of the running anastomotic suture.

**Fig. 3:**
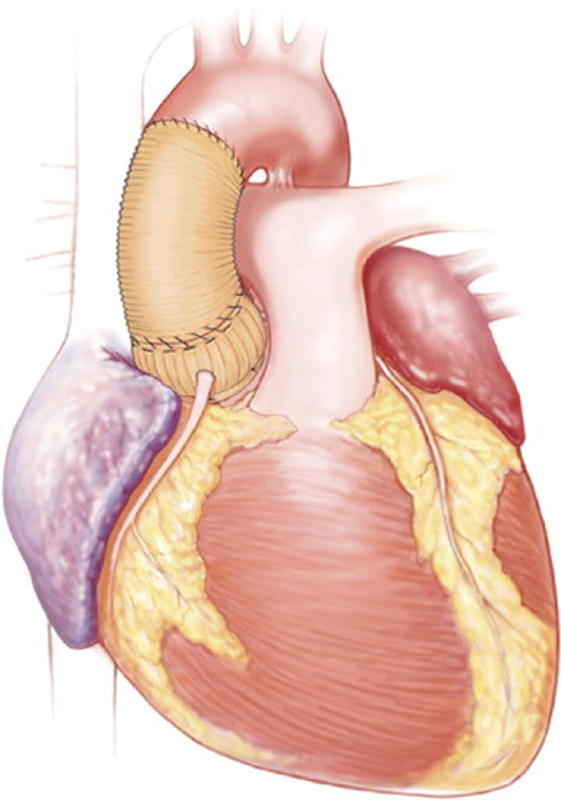
Completed repair.

Hess et al 8 9 have described details of the FL Sleeve procedure. Under cardioplegic arrest on cardiopulmonary bypass (CPB), we transected the ascending aorta immediately above the sinotubular junction. Typically, a total of 4 to 6 subannular mattress sutures were located in the same horizontal level up to 3 mm below the lowest point of the midpoint of the leaflets. Three of these mattresses were in the same line with valve commissures and another one was located under the noncoronary cusp ( Fig. 1 ). Hegar dilators (Jarit Instruments, Hawthorne, NY) or valve sizers were used for annular sizing. The main clinical goal was to ensure adequate leaflet coaptation and valve competence. The subannular sutures were placed through the sleeve graft and secured over a presized Hegar dilator to prevent narrowing of the annulus. With the sleeve graft temporarily seated, locations of the coronary arteries were marked on the graft, and vertical slits were made to create coronary keyholes. The slits were repaired using simple sutures below the coronary arteries after the sleeve graft was appropriately located over the aortic root. To prevent coronary artery impingement, intraoperative attention to the keyholes and intraoperative transesophageal echocardiography monitoring of ventricular function after CPB is imperative. The sleeve graft is secured at the sinotubular junction with a running horizontal mattress ( Fig. 2 ). To finalize the aortic root reconstruction, we used a smaller graft distal to the sleeve graft to reduce the sinotubular junction and incorporate the aorta and the sleeve graft via a hemostatic running suture ( Fig. 3 ).

### Patient Outcomes

The primary endpoints chosen were the procedural safety, long-term durability, and freedom from reoperation. Secondary endpoints included improvement of the aortic valve incompetency and left ventricular dimensions improvement. We used the following echocardiography measurements for comparison of perioperative and postoperative aortic valve and left ventricular functions: left ventricular end-diastolic diameter (LVEDD), left ventricular end-systolic diameter (LVESD), left ventricular ejection fraction (LVEF), and AI that was graded as: 0 = none, 1 = trace/minimal, 2 = mild, 3 = moderate, and 4 = severe. Follow-up echocardiography measurements were not available for all patients because some patients had postoperative follow-up echocardiography at outside centers. For patients who provided an informed consent (after March 2006), we called and sent a fax to the patients' PCP and local cardiologist to collect additional follow-up data, if available. We gathered patients' survival data and need for reoperation via social security death index and our electronic medical record database (EPIC), respectively, and verified their status with patients' PCPs or cardiologists to validate our data.

### Statistical Analysis

Continuous variables were presented as the mean ± standard deviation (SD) and categorical data as frequency and percentage. Student's *t* -test and Wilcoxon matched-paired test were used to compare the echocardiography measurements before and after the FL Sleeve procedure, if applicable. Patients' survival rate and freedom from reoperation were evaluated by Kaplan–Meier and life-table methods. All analyses were performed by SPSS software (Version 22, IBM Co., Armonk, NY).

## Results

### Patients' Demographics

One hundred and seventy-seven patients with mean age of 49.41 ± 15.37 years were included. Sixteen (9.03%) patients underwent the FL Sleeve procedure in an emergent status due to acute Type A aortic dissection and all others had surgery due to chronic dissection or aortic aneurysm. Thirty-seven (20.9%) patients had Marfan syndrome. Mean ± SD aortic diameter was 53.72 ± 7.85 mm ( Table 1 ). A majority of patients had concomitant cardiac surgery in addition to the FL Sleeve procedure ( *N* = 123, 69.49%) ( Table 2 ).

**Table 1:** Patients' preoperative characteristics

Variables a	Absolute values	Percentages (%)
Age (y)	49.41 ± 15.37	
Male	128	72.31
Aortic diameter (mm)	53.72 ± 7.85	
Hypertension	93	52.54
Diabetes	12	6.77
Prior stroke	9	5.08
Prior TIA	4	2.25
Prior myocardial infarction	1	0.56
Prior coronary artery bypass graft	3	1.69
Type A dissection	16	9.03
Marfan syndrome patients	37	20.91

Abbreviations: SD, standard deviation; TIA, transient ischemic attack.

a Continuous data are presented as mean ± SD or median (range) and categorical data as number (%).

**Table 2:** Intraoperative and postoperative outcomes

Variables a	Absolute values	Percentages (%)
Concomitant cardiac surgery	123	69.49
Arch and hemiarch reconstruction	79	44.63
Subcommissural annuloplasty	43	24.29
Coronary artery bypass	19	10.73
Pulmonary vein isolation	4	2.25
Maze procedure	2	1.12
Patent foramen ovale closure	3	1.69
Atrial septal defect repair	2	1.12
Mitral valve repair	2	1.12
Pulmonary valve replacement	1	0.56
Ligation of patent ductus, *N* (%)	1	0.56
*Perioperative times and events:*		
Cardiopulmonary bypass time (min)	180.79 ± 54.75	
ICU hours	102.30 ± 106.49	
Ventilation hours	20.37 ± 25.75	
Intraoperative blood transfusion	61	34.46
In-hospital myocardial infarction	0	0
Postoperative endocarditis	0	0
In-hospital Stroke/TIA	3	1.69
Reintervention due to bleeding	3	1.69
Length of stay (d) In-hospital death	8.76 ± 5.6 3	1.69
Readmission within 30 d	15	8.47
Reintervention in 30-d readmission	0	0
30-d mortality (after discharge)	0	0

Abbreviations: ICU, intensive care unit; SD, standard deviation; TIA, transient ischemic attack.

a Continuous data are presented as mean ± SD or median (range) and categorical data as number (%).

### Early Outcome

Three (1.69%) patients required reintervention due to bleeding. No patient developed postoperative endocarditis or myocardial infarction. Nine (5.08%) patients required new permanent pacemaker implantation. Three (1.69%) patients died during hospitalization. No patient died in the Type A dissection group. No other patients died after discharge within 30 days of the surgery. Three (1.69%) patients developed postoperative stroke during hospitalization. However, no other cerebrovascular event was detected within 30 days of surgery or later. Following discharge, 15 (8.47%) patients needed readmission within 30 days of surgery, but no intervention was required ( Table 2 ).

At 30 days, 140 (140/177, 79%) patients had postoperative follow-up echocardiography. No (0%) patient had severe AI at 30 days and only 3 (3/140, 2.14%) patients had moderate AI. Mean ± SD AI grade significantly decreased from baseline to 30 days, 2.18 ± 1.26 versus 1.1 ± 0.93 ( *p* < 0.001). LVEDD and LVESD also greatly decreased from 52.20 ± 6.73 to 46.87 ± 8.40 ( *p* < 0.0001) and from 35.73 ± 8.1 to 34.58 ± 8.7 at 30 days ( *p* = 0.05), respectively. However, mean ± SD LVEF did not noticeably change from baseline to 30 days, 57.92 ± 6.8 versus 55.55 ± 10.73 ( *p* = 0.10).

### Midterm Echocardiography Follow-Up

One patient developed severe AI and required AVR within the first year of operation. We had 41 patients with available follow-up echocardiography between 2 and 3 years. Preoperative mean ± SD AI grade decreased from 2.17 ± 1.35 at baseline to 1.17 ± 0.94 at midterm ( *p* < 0.001). Baseline mean ± SD LVEDD significantly decreased at midterm, 50.96 ± 6.80 versus 46.07 ± 6.40 ( *p* = 0.01). Mean ± SD LVESD also decreased from baseline to midterm, 35.48 ± 8.15 versus 30.88 ± 4.05 ( *p* = 0.002). However, improvement of LVEF was not significant, baseline: 57.93 ± 8.41 versus midterm: 58.15 ± 8.52 ( *p* = 0.89).

### Long-Term Follow-Up

**Fig. 4:**
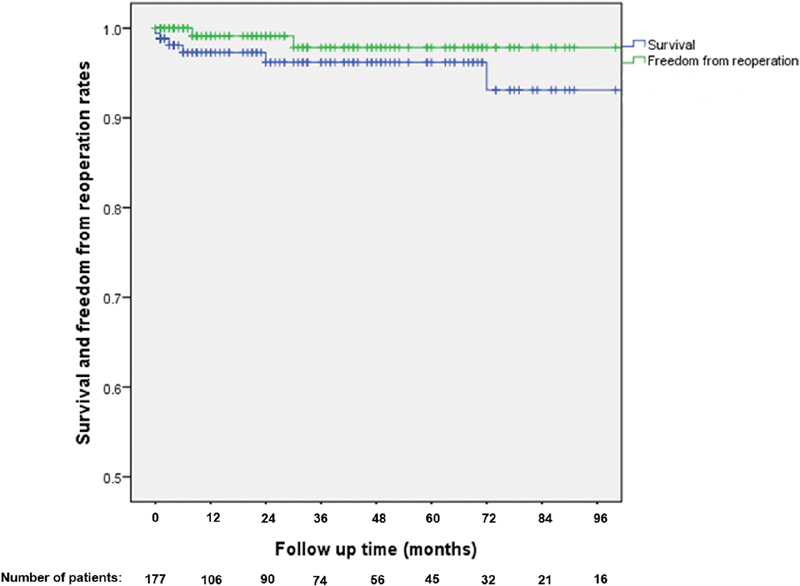
Patients' survival and freedom from reoperation rates after the Florida Sleeve procedure.

Clinical follow-up including patients' survival status and need for reoperation was completed for all patients. No patient developed aortic dissection during follow-up. Three (1.69%) patients needed reoperation; 1 (0.56%) patient with a bicuspid valve repair had AVR due to severe AI at 8 months, 1 (0.56%) patient had degeneration of distal aorta from a baseline Type A dissection repair and underwent endovascular aneurysm repair and thoracic endovascular aortic repair at 26 months, and then transcatheter AVR at 30 months due to central AI; and another (0.56%) patient with Marfan syndrome required ascending aorta replacement due to a pseudoaneurysm at 112 months. The mean ± SD duration of freedom from reoperation was 136.99 ± 45.56 months. We estimated patients' overall freedom from reoperation as 99% at 1 year and 98% at 2 to 8 years ( Fig. 1 ). Freedom from any type of aortic dissection was 100% at 1 to 8 years, whereas freedom from AVR was 99% at 1 year and 98% at 2 to 8 years. Patients had survival rate of 98% at 1 year, 97% at 5 years, and 93% at 8 years ( Fig. 4 ).

To perform subgroup analysis for patients who had both baseline and long-term (5 and 10 years) follow-up echocardiography, the patients with AVR at 8 and 30 months were excluded. At 5 years, 31 patients had follow-up echocardiography. Of these 31 patients, 8 (8/31, 25.80%) patients had moderate AI and 3 (3/31, 9.67%) others had severe AI preoperatively. Only 2 (2/31, 6.45%) patients remained with moderate AI at 5 years, and no patient with severe AI was identified. Freedom from severe AI was 100% and freedom from AI greater than mild (> 2 + ) was 93.6% at 5 years. AI grade significantly decreased from 2.00 ± 1.23 at baseline to 1.45 ± 0.88 at 5 years ( *p* = 0.04). Although preoperative mean ± SD LVEDD greatly decreased from 53.22 ± 7.07 to 46.11 ± 10.51 at 5 years ( *p* = 0.01), changes in mean ± SD of LVESD and LVEF were not significant from baseline to 5 years (from 35.23 ± 9.68 to 33 ± 9.40, *p* = 0.27 and from 57.80 ± 7.88 to 54.57 ± 13.76, *p* = 0.13, respectively). At 10 years, 11 patients had a follow-up echocardiography. Of these, 3 (3/11, 27.27%) patients had moderate AI and 2 (2/11, 18.18%) had severe AI at baseline. At 10 years, no patient had severe AI and only 1 (9.09%) patient remained in moderate AI. Because of the small number of patients and lack of statistical power, we did not compare baseline versus 10 years echocardiography measurements.

## Discussion

This retrospective study includes the largest number of patients who underwent the FL Sleeve procedure. In-hospital mortality rate was 1.69%, and 30-day mortality rate remained the same. The outcomes of the study, including patients' survival rate and freedom from reoperation, were excellent.

Two initial studies employing the David and Yacoub techniques had higher rates of early mortality compared to the present series, 4.8 and 4.6% versus 1.69%, respectively. 4 12 In contrast, two recent studies of the David and Yacoub procedures reported improved 30-day mortality rates (1–1.7%). 13 14 A recent report from Emory on the David procedure had a higher operative mortality at 5.7%. 15 However, Ouzounian et al 2 recently updated the Toronto experience with an in-hospital mortality (0.4%). The low rate of early mortality in their study is maybe related to less concomitant cardiac surgery, exclusion of nonelective patients, and a younger patient population. Moreover, the study was conducted in a center with the world's highest level of expertise for the David procedure. Given the complexity of the David procedure, a very low rate of early mortality may not be reproducible from all centers. On the other hand, Shrestha et al 12 did not have any 30-day mortality when they limited their patients to elective and isolated David procedure.

In an earlier report, Hess et al 9 reported a 6.66% early mortality rate in patients who underwent the FL Sleeve procedure at our center. The mortality rate (1.69%) in this series is lower than the previous report due to a larger number of patients and increased surgeons' experience. Type A aortic dissection has been suggested as a risk factor for operative mortality. 16 17 Coselli et al 14 reported intraoperative death in a patient with Type A aortic dissection during the David procedure, and Leshnower et al 17 found 4.7% operative death among these patients. Importantly, Leyh et al 18 reported Type A dissection repair with a Yacoub or David procedure with early mortality as high as 17%. In the present series of 177 FL Sleeve patients, there were 16 Type A dissections with no mortality.

The present study is the only study that has investigated long-term outcome of patients after the FL Sleeve procedure. In the previous study by Hess et al, 9 they reported four late deaths. However, the authors were unable to estimate patients' mid- or long-term survival rates. In this series, we found a 97% survival rate at 5 years and 93% survival rate at 8 years. Kvitting et al 19 examined previous AVS techniques and found a similar survival rate at 5 years (98.7%). In the study by the Hannover Medical School group, including 450 patients with the David procedure, 5-year survival was 85% and 10-year survival rate decreased to 70%. 12 David et al 20 reported 36 to 83% survival rate at 8 years' follow-up from David procedures. Coselli et al 14 employed the David procedure and reported a survival rate of 86.9% at 8 years. Importantly, no patients with acute aortic dissection died throughout our study and this cohort had 100% survival rate at 1 to 8 years. In a contrasting report, patients with acute aortic dissection who underwent a Yacoub procedure had 1, 5, and 10 years survival rates of 73, 63, and 53%. 4

In this FL Sleeve series, there was excellent overall freedom from reoperation at 8 years (98%). Kvitting et al 19 also found a high degree of freedom from reoperation at 10 years (92%) after the David V procedure. Liebrich et al 21 found 94% freedom from reoperation at 5 years and 90% freedom from reoperation at 10 years. Another study utilizing Yacoub's technique reported freedom from reoperation of 89% at 10 years. 4 David et al 22 also found 99% freedom from aortic valve reoperation at 5 years. In their more recent report from Ouzounian et al 2 and David et al, there was 100% freedom from aortic reoperation at 5 years and 97% freedom from aortic reoperation after 10 years. Long-term freedom from AVR in our study was higher than other studies with early reports on their AVS approaches (from 87 to 96%). 12 14 23

Gamba et al 11 found no severe AI and only 1% moderate AI after 18 ± 9 months of the sleeve procedure. On the other hand, Hess et al's FL Sleeve report has described improvement of AI at 1 week following the FL Sleeve procedure, but a trend toward increasing mean AI grades at 2 years, which raised doubt on the durable improvement of aortic valve function with the FL Sleeve procedure. 9 Although one bicuspid valve repair patient developed severe AI at 8 months and underwent an AVR, the present series confirms that the FL Sleeve procedure is effective in improving AI as more than 90% of our patients were free from greater than mild AI. A study by David et al 13 showed 90% freedom from moderate or severe AI at 5 years. Esaki et al's 15 recent series with the David procedure included 282 patients, and at 7 years, freedom from greater than moderate AI was estimated at 98%. On the other hand, in Yacoub et al's study, 4 17.7% of patients had severe AI prior to surgery and 3% of patients still had severe AI at 5 years. In our current study, 15.25% of patients had severe AI before the FL Sleeve procedure, but no patients remained in severe AI at 5 or 10 years.

Consistent with Hess et al's earlier report, 9 we found that the FL Sleeve procedure can improve left ventricular end-diastolic dimensions. Although LVEF did not change during the follow-up in our study and LVESD significantly improved at midterm follow-up only, improvement of LVEDD was noticeable from baseline to 30 days, midterm, and 5 years. One study of the David procedure did find significant improvement of LVEDD at 30 days, but the diameter increased again at 5 years. 5 Similar to our study, Yacoub et al 4 also had noticeable loss to follow-up in their first report with completed follow-up for only 5 patients that noted improvement of both LVESD and LVEDD.

There were 3 (1.69%) perioperative strokes, and no other cerebrovascular events were reported during follow-up. Yacoub et al 4 reported 7 (5.83%) perioperative strokes. David et al 13 15 reported 7 thromboembolic events in their first report, although the rate of stroke in the most recent study from David technique was only 3.3%. At least two other studies with the David procedure had only one postoperative stroke. 2 21 Shrestha et al 12 had no long-term stroke after the David I procedure and concluded that there is an extremely low risk of valve-related complications. Our findings confirm the risk of early and late thromboembolic events to be very low after the FL Sleeve procedure.

No patient developed aortic dissection during follow-up in this FL Sleeve series. Dissection was found in 2.6% of patients after the David procedure in Kvitting et al's study. The freedom from Type B aortic dissection was estimated to be 97.5% at 5 years and 90% at 10 years in their study. 19 Another study on AVS procedures found 88 and 84% freedom from aortic dissection at 5 and 10 years. 24 It is noteworthy that in our previous study of FL Sleeve outcomes in patients with Marfan syndrome, no patient developed aortic dissection during follow-up. 25

AVR including composite root replacement is associated with a higher rate of cardiac mortality and valve-related morbidity versus AVS techniques. 2 The David valve-sparing procedure has been suggested as the “gold standard” for patients with aortic root dilation, 5 but in many centers adoption of AVS procedures has remained around 15%. On the other hand, due to complexity of the David procedure, Ouzounian et al 2 recommended that patients should be referred to large centers with highly experienced surgeons. However, even in a multicenter trial of highly experienced surgeons, 7% of patients developed greater than mild (2 + ) AI at 1 year. 26 We believe that valve-sparing procedures could be available in more centers with the simplified FL Sleeve procedure. We are encouraged that the outcomes of the FL Sleeve procedure are excellent, although further study is warranted.

### Limitations

We recognize the following limitations in our study: (1) a retrospective and single-center study, (2) no comparison between outcomes of FL Sleeve procedure and previous AVS techniques, and (3) loss to follow-up of patients' echocardiography measurements.

## Conclusion

The Florida Sleeve technique is a safe, effective, and durable procedure for aortic root dilation and functional aortic insufficiency. Patients' long-term survival, freedom from reoperation, and freedom from significant AI are excellent. Owing to the simplicity and reproducibility of the FL Sleeve procedure, it can be an appropriate alternative valve-sparing procedure.
